# Upregulation of MiR-205 transcriptionally suppresses SMAD4 and PTEN and contributes to human ovarian cancer progression

**DOI:** 10.1038/srep41330

**Published:** 2017-02-01

**Authors:** Juanni Li, Kuan Hu, Guanghui Gong, Ding Zhu, Yixuan Wang, Hailing Liu, Xiaoying Wu

**Affiliations:** 1 Department of Pathology, Xiangya Hospital, Central South University, Changsha, Hunan, China; 2 Department of Pathology, School of Basic Medical Science, Central South University, Changsha, Hunan, China; 3 Department of Hepatobiliary Surgery, Xiangya Hospital, Central South University, Changsha, Hunan, China

## Abstract

MicroRNAs (miRNAs) function as critical regulators of gene expression and their deregulation is associated with the development and progression of various cancers. This study aimed to investigate the biological role and mechanism of miR-205 in ovarian cancer (OC). MiR-205 was upregulated in OC tissues and cells in comparison to the controls. Meanwhile, overexpression of miR-205 was significantly associated with poor overall survival of OC patients. Functional study indicated that ectopic expression of miR-205 significantly promoted cell proliferation, migration, invasion and chemoresistance of OC cells. SMAD4 and PTEN were identified as direct targets of miR-205 using luciferase reporter assays, real-time PCR (qRT-PCR), and western blot. Most interestingly, *in vivo* studies indicated that miR-205 markedly promoted the growth and metastasis of tumors and the expression of miR-205 was also found to be inversely correlated with that of SMAD4 and PTEN in nude mice. Overall, we suggest that miR-205 functions as an oncogenic miRNA by directly binding to SMAD4 and PTEN, providing a novel target for the molecular treatment of ovarian cancer.

Ovarian cancer is a gynecological malignancy with high mortality rates worldwide^1^. Recently, Epithelial ovarian cancer (EOC), which accounts for ~90% of all ovarian cancers, became the most lethal gynecological cancer and the fifth leading cause of cancer-related death in women^2^,^3^. Despite lots of improvements in therapeutic strategies and surgical techniques, the prognosis of ovarian cancer still remains poor, largely attributed to lack of early, safe detection methods and the high failure rate for chemotherapy^4^. In order to improve the survival of patients with ovarian cancer, it is important to explore the key molecular mechanisms of EOC initiation and development and to identify the relevant factors involved in metastasis and chemotherapy^5^,^6^,^7^.

Smad4, a member of the Smad family, is a central molecule of TGF-β signaling pathway. Smad4 form a complex with phosphorylated Smad2/Smad3 makes it possible for TGF-β executing its biological activity^8^,^9^, which is associated with cellular adhesion, motility, differentiation, and so on. During cancer development, lost or reduced expression of Smad4 is frequently found. For example, in pancreatic cancer, loss of Smad4 is observed and leads to the switch of TGF-β from a tumor-suppressive to a tumor-promoting pathway through its interaction with E-cadherin, vimentin and beta-catenin^10^. In ovarian cancers, down-regulated Smad4 was detected in clinical patients specimens, which indicated that Smad4 might enhance TGF-β signaling^11^. However, the mechanism of smad4 expression regulation is still unclear.

PTEN, a wellknown tumor suppressor located in 10q23.3, is the only lipid phosphatase known to counteract the PI3K pathway and plays an important role during cancer development^12^. Many researchers have confirmed its ability to block tumor growth and chemoresistance through inhibiting multiple cell signaling pathway^13^. Loss of PTEN expression represents a common event in various of tumor types, indicating that low PTEN expression is a critical factor in promoting cancer development. However, the role of PTEN in OC remains unclear.

MiRNAs are short noncoding RNAs with about 22 nucleotides in length which suppress gene expression via direct binding to the 3′ untranslated region (UTR) of mRNAs, leading to mRNA degradation or post-transcriptional translational repression^14^,^15^,^16^,^17^. Growing evidence has demonstrated that miRNAs are involved in various biological processes such as cell proliferation, migration, invasion, differentiation, and so on^18^,^19^,^20^,^21^. In cancer, miRNAs can behave as oncogenes or tumor suppressor genes depending on the cellular function of their target^22^. Therefore, better understanding the role of miRNA during ovarian carcinogenesis and metastasis may provide new avenues for OC diagnostic and treatment regiments.

In this study, we investigated the potential roles and related target genes of miR-205 in OC via a series of experiments *in vitro* and *in vivo*. We reported that miR-205 was upregulated in OC tissue specimens and cell lines, and significantly correlated with a poor prognosis in OC patients. Ectopic miR-205 promoted the proliferation, migration, invasion and chemoresistance of ovarian cancer cells *in vitro*. Furthermore, we demonstrated that miR-205 directly targeted and downregulated SMAD4 and PTEN via recognizing their 3′ UTRs. Animal studies showed that miR-205 markedly promoted the growth and metastasis of tumors. Altogether, these data demonstrate that miR-205 plays an important role in the development and progression of ovarian cancer and may represent as a potential therapeutic target for ovarian cancer.

## Results

### MiR-205 is upregulated in OC and correlated with poor survival

**Figure 1:**
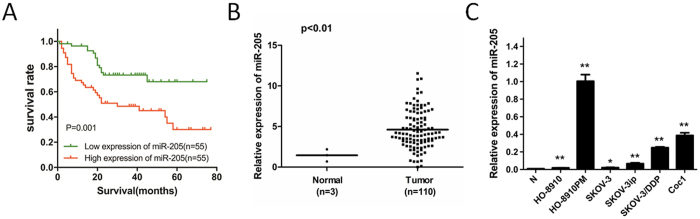
miR-205 is upregulated in ovarian cancer. (**A**) Kaplan-Meier analysis indicates upregulation of miR-205 was significantly associated with poorer overall survival rates of OC patients (P = 0.001). (**B**) qRT-PCR shows that miR-205 expression was frequently upregulated in 110 OC tissues compared with three normal ovarian tissues. (P < 0.01). (**C**) Upregulation of miR-205 was detected in all 6 OC cell lines compared with pool of normal ovarian tissues (N). U6 was set as an endogenous control. The results were presented as means ± SEM (n = 3 for each panel). Statistical significance was concluded at *P < 0.05, **P < 0.01, ***P < 0.001.

We detected miR-205 expression in 110 archived clinical ovarian cancer tissues. Kaplan-Meier analysis indicated that the 5-year overall survival (OS) rates of ovarian cancer patients with high miR-205 expression was significantly lower (Fig. 1A). Next, to verify the high expression of miR-205 in ovarian cancer patients, miR-205 expression of 110 ovarian cancer tissues paired with three normal ovarian tissues were detected by qRT-PCR. The result showed that miR-205 was significantly overexpressed in ovarian cancer tissues (Fig. 1B). In agreement with these observations, upregulation of miR-205 was also confirmed in 6 ovarian cancer cell lines (HO-8910, HO-8910PM, SKOV-3, SKOV-3ip, SKOV-3/DDP, and COC1) compared with a pool of 3 normal ovarian tissues as a normal control (Fig. 1C). Taken together, these results strongly suggest that miR-205 is correlated with poor prognosis, and is upregulated in ovarian cancer, suggesting that miR-205 functions as an oncogene in ovarian cancer development.

### MiR-205 promotes OC cell proliferation, migration and invasion *in vitro*

**Figure 2:**
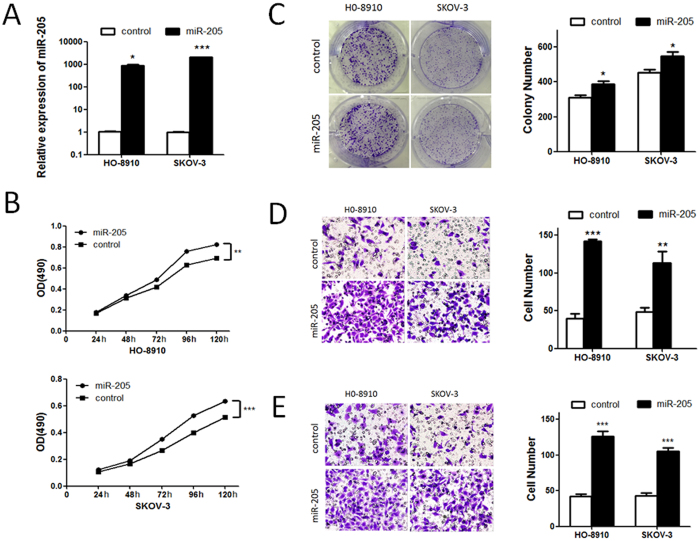
miR-205 promotes OC cell proliferation, migration and invasion in OC cell lines. (**A**) miR-205 levels in HO-8910 and SKOV-3 cells stably transfected with LV-miR-205 or LV-miR-Ctrl. (**B**) The CCK-8 assay was used to measure the effect of miR-205 on OC cell growth. (**C**) The effect of miR-205 on colony formation in HO-8910 and SKOV-3 cells. (**D**) We performed the transwell migration assay to explore the effect of miR-205 on the migration of OC cells. (**E**) Effects of miR-205 on cell invasion in indicated cells *in vitro* using transwell matrigel assay. All the *in vitro* experiments were performed in triplicates and repeated three times. The results were presented as means ± SEM. *p < 0.05; **p < 0.01; ***p < 0.001 compared to controls.

Having observed the association of miR-205 expression and poor survival in OC patients, we set out to functionally characterize the effects of miR-205 on ovarian cancer cells. We infected HO-8910 and SKOV-3 cells with lentiviral vector harboring miR-205 (LV-miR-205) to establish two stably expressing miR-205 cells. Empty vector (LV-miR-Ctrl) transfected cells were used as controls. All these cell lines can stably express the firefly luciferase gene. The transfection efficiency was verified by qRT-PCR (Fig. 2A). We then used the Cell Counting Kit-8 (CCK-8) assay to assess the effects of miR-205 on cell proliferation, the result showed that miR-205 overexpression significantly increased the growth rates of HO-8910 and SKOV-3 cells (Fig. 2B), and the promoting effect of miR-205 on cell proliferation was further confirmed by colony formation assay (Fig. 2C). Next, we investigated the potential effect of miR-205 on cell motility and invasiveness. The migration assay demonstrated that HO-8910 and SKOV-3 cells overexpressing miR-205 were found to have significantly higher rate of migration than control cells (Fig. 2D). Similarly, the Matrigel invasion assay indicated that overexpression of miR-205 significantly promoted the invasiveness of HO-8910 and SKOV-3 cells (Fig. 2E). Collectively, these data clearly show that miR-205 is a promoter of proliferation, migration and invasion in OC cells.

### MiR-205 promotes OC cell chemoresistance

**Figure 3:**
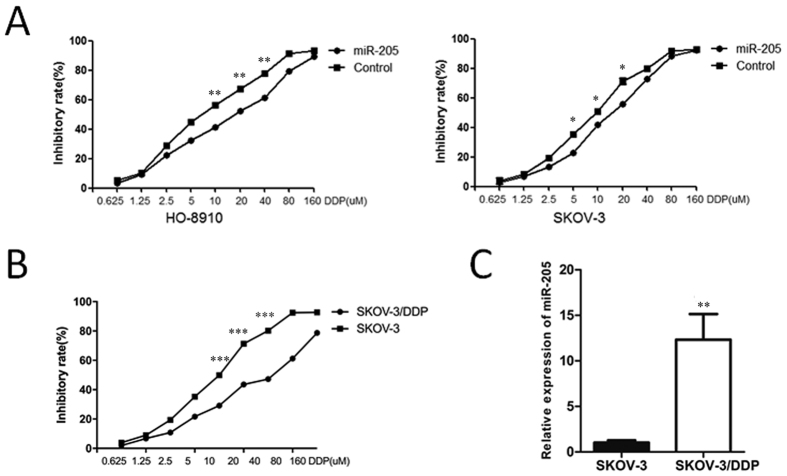
miR-205 promotes the chemoresistance of OC cells. (**A**) Effects of ectopic overexpression of miR-205 on the cisplatin chemoresistance of HO-8910 and SKOV-3 cells, as analyzed by the SRB assay. (**B**) The sensitivity of SKOV-3/DDP and SKOV-3 cells to cisplatin was measured using SRB assay. (**C**) The miR-205 expression level in SKOV-3/DDP and SKOV-3 cells was measured using qRT-PCR assay. The results were presented as means ± SEM; *P < 0.05, **P < 0.01, ***P < 0.001.

The effect of miR-205 on the sensitivity of OC cells to chemotherapeutic agent, cisplatin, was investigated. Overexpression of miR-205 led to an obvious increase in the IC50 value of cisplatin in both HO-8910 (IC50; miR 15.69 ± 1.05 control 7.35 ± 1.04) and SKOV-3 cells (IC50; miR 14.87 ± 1.03 control 9.22 ± 1.03) when compared with that in the control group (Fig. 3A). To further verify the promoting effect of miR-205 on cell chemoresistance, we also detect miR-205 expression in the cisplatin-resistant SKOV-3/DDP cells and its parent cancer cells SKOV-3. First, using the sulforhodamine B (SRB) assay, we proved that SKOV-3/DDP cells were indeed significantly more resistant to the therapy of cisplatin compared with SKOV-3 (IC50; SKOV-3/DDP 35.67 ± 1.06 SKOV-3 9.27 ± 1.03) (Fig. 3B). After this, miR-205 expression was further found to be higher in SKOV-3/DDP cells compared with that in SKOV-3 (Fig. 3C). This data thus prove that miR-205 promotes chemoresistance of OC cells.

### SMAD4 and PTEN are direct target of miR-205 in OC

**Figure 4:**
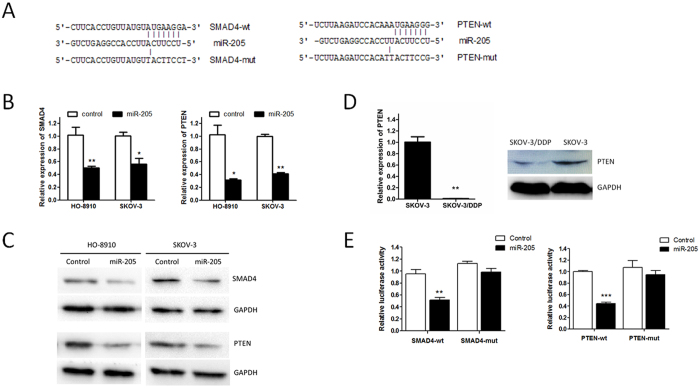
SMAD4 and PTEN are direct transcriptional targets of miR-205 in OC cells. (**A**) Putative target sites for miR-205 in the 3′ UTR of SMAD4 and PTEN. Target sequences of SMAD4- and PTEN-3′ UTR were mutated. (**B**) Effects of miR-205 upregulation on endogenous SMAD4 and PTEN mRNA level which were analyzed by qRT-PCR. (**C**) Decreased protein level of SMAD4 and PTEN due to miR-205 overexpression in HO-8910 and SKOV-3 cells, GAPDH served as a loading control. (**D**) qRT- PCR and western blotting detection of PTEN expression in SKOV-3/DDP and SKOV-3 cells. (**E**) Analysis of luciferase activity. The 3′ UTR wild-type report plasmid or 3′ UTR mutant report plasmid of SMAD4 or PTEN were transfected into 293 T cells, and LV-miR-205 or LV-miR-Ctrl was co-transfected with each plasmid in 293 T cells. After which the luciferase activity was analyzed using the dual-luciferase report assay system. All the error bars indicate ± SEM. Statistical significance was concluded at *P < 0.05, **P < 0.01, ***P < 0.001.

It has been demonstrated that various biological and pathological processes, including tumor growth, cell invasion, and tumor metastasis, are regulated by small regulatory non-coding RNAs consisting of approximately 19–25 nucleotides; e.g. miRNAs^23^. Therefore, we hypothesized that some tumor suppressor genes targeted by miR-205 are downregulated in OC as miR-205 was upregulated in OC. Thus, to predict potential miR-205 targets, we used the miRWALK (http://zmf.umm.uni-heidelberg.de/apps/zmf/mirwalk2/), a comprehensive database on miRNAs with several established program; e.g. miRDB, miRanda, TargetScan, RNA22, and so on^24^. From the miRWALK, hundreds of potential targets were found to be targeted by miR-205. Among these genes, 14 tumor-suppressor genes were identified after reading literatures and then screened in our two stably expressing miR-205 cell lines (Supplementary Figure S1). Finally, SMAD4 and PTEN were selected for further study since these two genes were found to be the lowest expression in both HO-8910 and SKOV-3 cells overexpressing miR-205, and also have been reported to be down-regulated in OC previously and is closely involved in OC cell proliferation, migration, invasion and chemoresistance. Both SMAD4 and PTEN mRNAs had one possible binding site for miR-205 (Fig. 4A). First, endogenous SMAD4 and PTEN mRNA or protein levels were markedly decreased in HO-8910 and SKOV-3 cells stably expressing miR-205 compare to that of control cells (Fig. 4B,C). Consistent with above results, there was also a negative correlation between miR-205 expression and mRNA or protein levels of PTEN in SKOV-3/DDP and SKOV-3 cells (Fig. 4D). To confirm that SMAD4 or PTEN was directly inhibited by miR-205, a dual-luciferase reporter system was used. The wild-type or mutated 3′ UTR of SMAD4 and PTEN mRNA were inserted downstream of the luciferase reporter gene, and LV-miR-205 was co-transfected with each construct in HEK-293T cells. MiR-205 inhibited the firefly luciferase reporter activity of wild-type SMAD4 and PTEN 3′ UTR, but this inhibition was less changed for 3′ UTR with mutated binding sites (Fig. 4E). Taken together, these data indicate that miR-205 can repress the expression of SMAD4 or PTEN in OC cells by directly targeting the 3′ UTR of SMAD4 or PTEN mRNA.

### MiR-205 promotes OC tumorigenesis *in vivo*

**Figure 5:**
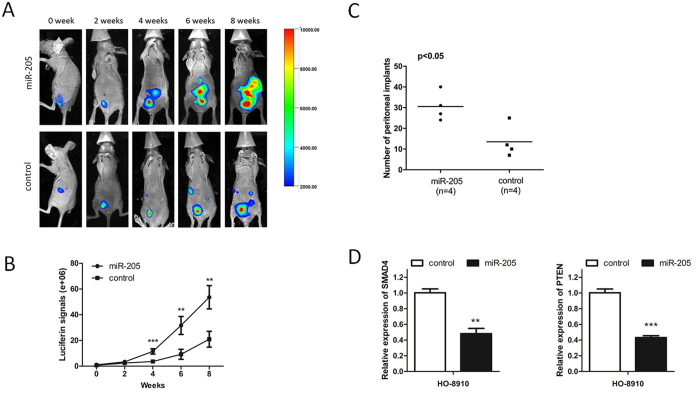
miR-205 promotes OC tumorigenesis *in vivo*. (**A**) Representative pictures of animals at different time points between 0 week and 8 weeks (from left to right) after inoculation with HO-8910 cells. Pictures were taken using an IVIS imaging system with i.p. injection of 100 mg/kg of D-luciferin. Color bars represented tumor cell intensity from low (blue) to high (red). (**B**) Luciferase activities of peritoneal tumors were measured every two weeks. The mice group that stably overexpressing miR-205 expressed significantly higher luciferase luminescence activities. (**C**) all the mice were sacrificed at the end of 8 weeks and the number of peritoneal implants were counted. Overexpression of miR-205 significantly promoted tumor dissemination. (**D**) SMAD4 and PTEN mRNA expression were significantly reduced in the miR-205 overexpression group compared with the control group. All the error bars indicate ± SEM; *P < 0.05, **P < 0.01, ***P < 0.001.

We then examined the tumor promotive role of miR-205 in ovarian cancer progression using an *in vivo* tumor model. Eight mice were randomly divided into two groups. HO-8910 cells stably expressing miR-205 or control cells were injected intraperitoneally (i.p.) into nude mice in each group. To assess peritoneal tumor progression over time, the luciferase activity was detected every two weeks. We found that four weeks after injection, the luciferase activity levels (reflecting tumor volume in the peritoneal cavity) was significantly higher in LV-miR-205 group compared to the LV-miR-Ctrl group (Fig. 5A,B and Supplementary Figure S2). In addition, all the mice were euthanized at the end of 8 weeks and lots of tumors on omentum, peritoneal surface, bowel mesentery, liver, ovary were observed in mice peritoneal cavity. We counted the number of peritoneal implants and found that the implants number in LV-miR-205 group were significantly more than that in LV-miR-Ctrl group (Fig. 5C). Furthermore, qRT-PCR analysis of the implanted tumors showed that SMAD4 and PTEN mRNA expression were significantly reduced in the LV-miR-205 group compared with the LV-miR-Ctrl group (Fig. 5D), further supporting the notion that miR-205 promoted OC tumorigenesis via SMAD4 and PTEN.

## Discussion

Tumor fast growth, migration, invasion, and chemoresistance have been identified as classical hallmarks of cancer malignancy, and are major causes of a poor prognosis in cancer patients^25^,^26^. Our study is the first to show that miR-205 functions as a tumor-promotive miRNA through directly binding to SMAD4 and PTEN in OC. We verified this finding by providing the following evidence. (1) miR-205 was upregulated in OC tissues and cells in comparison to the controls. (2) the overexpression of miR-205 was significantly associated with poor overall survival in clinical OC patients. (3) ectopic expression of miR-205 significantly promoted cell proliferation, migration, invasion and chemoresistance of ovarian cancer cells. (4) overexpression of miR-205 reduced mRNA and protein levels of SMAD4 and PTEN which were further identified as potential target genes of miR-205 by bioinformatics prediction and luciferase reporter assays. (5) *In vivo* studies demonstrated that miR-205 markedly promoted the growth and metastasis of tumors in nude mice. 6) mice with higher miR-205 expression had lower expression of SMAD4 and PTEN than that of controls. These finding suggest that miR-205 may play an important role in promoting carcinogenesis and chemoresistance of OC.

Ovarian cancer is the most common cause of death among gynecological malignancies, and it has a high mortality rate and low 5-year survival rate. Metastasis and chemoresistance are significant factors in the prognosis of OC^27^,^28^. It is well-known that genetic susceptibility, virus infection and environmental factors play important roles in ovarian cancer pathogenesis, however, the molecular mechanism for its development and progression remains unclear^29^,^30^. Therefore, it’s urgent to understand the molecular mechanisms underlying OC progression and identify novel avenues for targeted therapy. Recently, an increasing number of studies have reported that miRNAs have the potential to play a vital effect on ovarian cancer development.

Many miRNAs have been shown to behave as oncogenes or tumor suppressor genes depending on the cellular function of their targets^31^. MiR-205 is highly conserved in different kinds of lines and has been found to have close relationships with a variety of tumors. In bladder cancer, miR-205 was found to be significantly upregulated compared to normal tissue^32^. Moreover, High expression of miR-205 was also found in cervical cancer cell lines as well as clinical patient samples^33^. In contrast, low levels of miR-205 is observed in head and neck squamous cell carcinomas and associated with increased recurrence and poor prognosis^34^. Moreover, another study showed that miR-205 was a suppressor of prostate cancer development, and loss of miR-205 was associated with prostate cancer progression^35^. To our knowledge, the role and mechanism of miR-205 in OC have not been completely understood. Our data showed that miR-205 was upregulated in OC tissues and cells in comparison to the controls, and miR-205 overexpression was significantly associated with poor overall survival in OC patients. Furthermore, overexpression of miR-205 could promote cell proliferation, migration, invasion and chemoresistance of ovarian cancer cells. These finding suggested that miR-205 might act as an oncogene whose upregulation contributed to the progression and metastasis of OC.

However, the mechanisms underlying how miR-205 affects tumor progression were not clear. Therefore, we predicted potential targets of miR-205 by bioinformation analysis and several target genes were identified. Among these genes, SMAD4 and PTEN were selected for further study. Smad4 is a key mediator of TGF-β pathway^36^. Binding of the TGF-β to its receptor leads to phosphorylation of SMAD2/3 to form a transcription complex with SMAD4, this complex then binds to specific DNA sequences and regulates the expression of target genes that cause cell cycle arrest and apoptosis of epithelial cells^37^. PTEN is a key mediator of the PI3K/AKT pathway, and can catalyze the conversion of the membrane lipid second messenger PIP3 to PIP2. The downregulated PTEN upregulates PI3K/AKT signaling enhancing cell proliferation, migration and chemoresistance^38^. These information suggesting that oncogenic activity of miR-205 is possible, in part, through targeting SMAD4 and PTEN. In accordance with this hypothesis, we found that ectopic expression of miR-205 decreased SMAD4 or PTEN both mRNA and protein levels in OC cells. Furthermore, luciferase reporter assay showed that miR-205 reduced the luciferase reporter activity of wild-type 3′ UTR but not mutant 3′ UTR of SMAD4 or PTEN.

It is well-known that the poor 5-year overall survival in ovarian cancer are partly due to the development of platinum resistance^39^. Until now, DDP chemoresistance still remains a major obstacle for the successful treatment of ovarian cancer. Recently, an increasing number of studies have reported that miRNAs were involved in the process of cisplantin resistance in ovarian cancer^40^. In our study, we demonstrated that overexpression of miR-205 led to an obvious cisplatin resistance in OC cells and is negatively correlated with PTEN expression. Furthermore, the cisplatin-resistant OC cells SKOV-3/DDP showed a significant higher expression of miR-205, and lower PTEN expression compared with its parent cancer cells SKOV-3. Therefore, these findings showed that miR-205 could be a new member of the miRNAs that were involved in ovarian cancer cisplantin resistance, which was involved in AKT signal pathway through targeting PTEN. But it is unclear whether and how these confirmed miRNAs such as miR-205, miR-494, miR-214 directly targeting PTEN co-regulate the PTEN/Akt pathway, further effects the DDP resistance and other biological function in a collaborative or antagonistic manner.

To our knowledge, there is no study on miR-205 and OC tumorigenicity in an animal model. In our study, the tumor-promotive role of miR-205 *in vivo* was treated by intraperitoneally injecting HO-8910 cells stably expressing miR-205 and monitored by IVIS system. Further qRT-PCR analysis indicated the negative regulation of miR-205 to SMAD4 or PTEN.

In summary, this study identified miR-205 as a novel oncogenic miRNA that is stimulated in both *in vitro* and *in vivo* studies in OC. MiR-205 was upregulated in ovarian cancer and overexpression of miR-205 promoted ovarian cancer cell proliferation, metastasis both *in vitro* and *in vivo*. Overexpression of miR-205 also increased the chemoresistance of OC cells. Furthermore, the function of miR-205 in ovarian cancer may be exerted via downregulation of the target genes SMAD4 and PTEN, which play an important role in the function of miR-205 in ovarian cancer. Therefore, this research indicates that miR-205 may play an important role in ovarian cancer progression and could be targeted for the development of novel treatment for OC in the future.

## Methods

### Patients and samples

Written informed consent was obtained from all patients prior to the study, and this study was approved by the Ethics Review Committee of Xiangya Hospital of Central South University and the Ethics Review Committee of the Affiliated Cancer Hospital of XiangYa School of Medicine and conducted according to all current ethical guidelines. The 110 paraffin-embedded, archived ovarian cancer tissues used in this study were histopathologically and clinically diagnosed at the Xiangya Hospital and the Affiliated Cancer Hospital of XiangYa School of Medicine between 2008 and 2011. Three fresh normal ovarian tissues were each collected from three patients, and were frozen and stored in liquid nitrogen until used. All specimens had been confirmed with pathological diagnosis.

### Cell lines and cell culture

Epithelial ovarian cancer cell lines HO-8910 and SKOV-3 were purchased from CCTCC (China Center for Type Culture Collection) and CRC/PUMC (Cell Resource Center, IBMS, CAMS/PUMC), respectively. Epithelial ovarian cancer cell lines HO-8910PM and SKOV-3ip were kindly donated by Professor Xin Lu (Obstetrics and Gynecology Hospital, Fudan University, Shanghai, China). SKOV-3/DDP (cisplatin-resistant SKOV-3) cells were kindly donated by Professor Xiong Li (Xiangya Medical School, Central South University, Changsha, China). COC1 cells were kindly donated by Professor Bilian Jin (Institute of Cancer Stem Cell, Dalian Medical University, Dalian, China). All cell lines were cultured using standard protocols and incubated at 37 °C under a 5% CO_2_ humidified atmosphere.

### Establishment of stably expressing miR-205 ovarian cancer cell lines

The lentivirus vectors containing firefly luciferase, LV-hsa-miR-205 or LV-hsa-miR-Ctrl, were obtained from GENECHEM (Shanghai, China). HO-8910 and SKOV-3 cells were used as follows: 2 × 10^4^ cells were plated in 12-well plates overnight, and then 10 μL of lentivirus (2 × 10^8^ IU/mL) diluted in 1 mL of fresh 1640 medium supplemented with 10% FBS was added. 24 hours later, we replaced the culture medium with fresh 1640 medium. 3 days later, 1 μg/mL puromycin (Sigma-Aldrich, St. Louis, MO, USA) was added to the medium and replenished every two days for two weeks to select the cells transfected with the lentivirus.

### RNA extraction and quantitative real-time PCR (qRT-PCR) analyses

Total RNA was isolated from cell lines and FFPE tissue slides using Trizol reagent (Invitrogen) and the miRNeasy FFPE Kit (Qiagen, Venllo, The Netherlands), respectively. For the detection of miR-205, RNA was reverse transcribed into cDNA using All-in-One™ miRNA First-Strand cDNA Synthesis Kit (GeneCopoeia, Guangzhou, China), and then was used to perform qRT-PCR using All-in-One miRNA qRT-PCR Detection Kit (GeneCopoeia, Guangzhou, China). For the detection of mRNAs, RNA was reverse transcribed into cDNA using the the GoScript Reverse Transcription (RT-PCR) System (Promega, Madison, WI, USA), and the qRT-PCR for mRNAs was performed using GoTaq® qPCR Master Mix (Promega, Madison, WI, USA). All qRT-PCR was performed using ABI Prism 7500 system. U6 or GAPDH was used as an internal control for microRNA and mRNA quantification respectively. The primer sequences for the qRT-PCR are listed in Supplementary Table S1.

### Cell proliferation and colony formation assays

The cell proliferation assay was performed using the Cell Counting Kit-8 (CCK-8, Dojindo, Japan) according to the manufacturer’s instructions. For the colony formation assay, the methods were described previously^41^.

### Cell migration and invasion assay

The methods were described previously^41^.

### Drug sensitivity analysis

We examined the relationship between miR-205 expression and cell sensitivity to a chemotherapeutic drug, Cisplatin (Sigma-Aldrich, St. Louis, MO, USA). Cytotoxicity of Cisplatin was measured using the sulforhodamine B (SRB) assay. The cells were plated in 96-well plates with 0.625, 1.25, 2.5, 5, 10, 20, 40, 60, 80, or 160 uM of Cisplatin and the percentage of inhibitory was calculated after 48 h Cisplatin treatment.

### Western blot analysis

Equal quantities of denatured protein samples were separated by SDS-PAGE gels and transferred onto PVDF membranes. After blocking with 5% non-fat milk, the blots were incubated with the following primary antibodies: anti-SMAD4 (Santa Cruz Biotechnology, CA, USA), anti-PTEN (Cell Signaling Technology, Danvers, MA) followed by horseradish peroxidase-conjugated secondary antibody (Santa Cruz Biotechnology, CA, USA). Protein expression levels were detected using Image Lab software (Bio-Rad, CA, USA) and Anti-GAPDH (Santa Cruz Biotechnology, CA, USA) was used as a loading control.

### Luciferase activity assay

The PsiCHECK-2-SMAD4-wt and PsiCHECK-2-SMAD4-mut vectors or PsiCHECK-2-PTEN-wt and PsiCHECK-2-PTEN-mut vectors were purchased from Promega (Madison, WI, USA). 293 T cells were cultured in 24-well plates and co-transfected with 3′ UTR wt plasmid or 3′ UTR mut plasmid in the presence of either miR-205 or negative control. After 48 h, Luciferase and Renilla signals were measured using a Dual Luciferase Reporter Assay Kit (Promega, Madison, WI, USA) according to the manufacturer’s protocol.

### Animal model studies

All animal experiments were conducted according to the protocols approved by the Animal Care and Use Committee at Central South University, China. The mice (female 4–5 week old, 18~20 g BALB/c nude mice) were randomly assigned to two groups (n = 4/group). The HO-8910 cells (1 × 10^6^ cells) stably expressing either LV-miR-205 or LV-miR-Ctrl were injected intraperitoneally (i.p.) into two groups. For bioluminescence imaging of living animals, mice were injected i.p. with 100 mg/kg D-luciferin (Caliper Life Sciences, Hopkinton, MA, USA) in PBS. Then around 3 minutes later, mice were anesthetized with 2.0% isofluorane, and imaged by IVIS system (Bruker, Billerica, MA, USA) every two weeks. All the mice were euthanized at the end of 8 weeks and the peritoneal nodules were counted and collected.

### Statistical analysis

Statistical analyses were carried out using SPSS, version 18.0 (SPSS, Inc, Chicago, USA). Survival curves were plotted by the Kaplan-Meier method and compared by the log-rank test. The difference among groups was determined by the 2-tailed Student’s *t*-test. P values < 0.05 was considered statistically significant.

## Additional Information

**How to cite this article**: Li, J. *et al*. Upregulation of MiR-205 transcriptionally suppresses SMAD4 and PTEN and contributes to human ovarian cancer progression. *Sci. Rep.* **7**, 41330; doi: 10.1038/srep41330 (2017).

**Publisher's note:** Springer Nature remains neutral with regard to jurisdictional claims in published maps and institutional affiliations.

## Supplementary Material

Supplementary Figures and Tables
