# Supplementary material for: Upregulation of MiR-205 transcriptionally suppresses SMAD4 and PTEN and contributes to human ovarian cancer progression
Source: Sci Rep. 2017 Feb 1;7:41330. doi: 10.1038/srep41330 (PMC5286557; doi:10.1038/srep41330)
Supplement: Supplementary Figures and Tables [file srep41330-s1.pdf]

Upregulation of MiR-205 transcriptionally suppresses SMAD4 and PTEN and contributes to human ovarian cancer progression

Juanni Li[1,2], Kuan Hu[3], Guanghui Gong[1,2], Ding Zhu[1,2], Yixuan Wang[1,2], Hailing Liu[1,2], Xiaoying Wu[1,2,]*

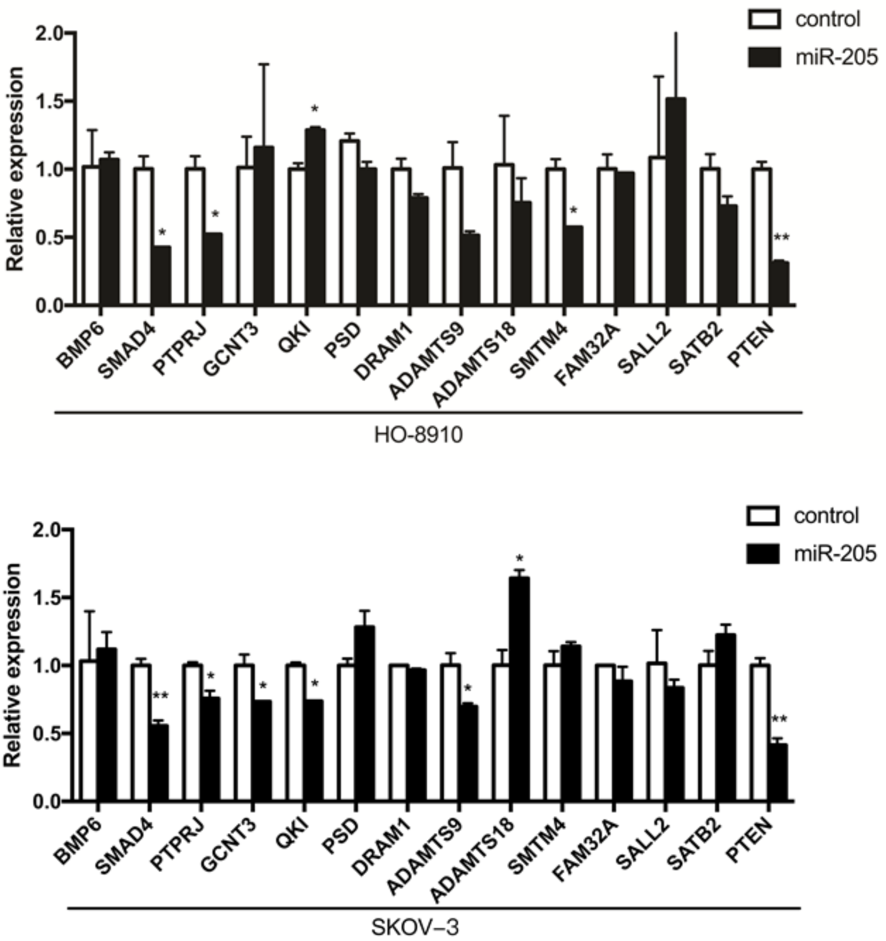

Supplementary Figure 1. Several miR-205 predicted target genes expression in two OC stable cell lines. qRT-PCR analysis of 14 miR-205 candidate targets expression in both HO-8910 and SKOV-3 cells stably expressing miR-205 and control cells. Transcript levels were normalized to GAPDH expression. Experiments were repeated at least three times with similar results; values are mean ± SEM; *P < 0.05, **P < 0.01, ***P < 0.001.

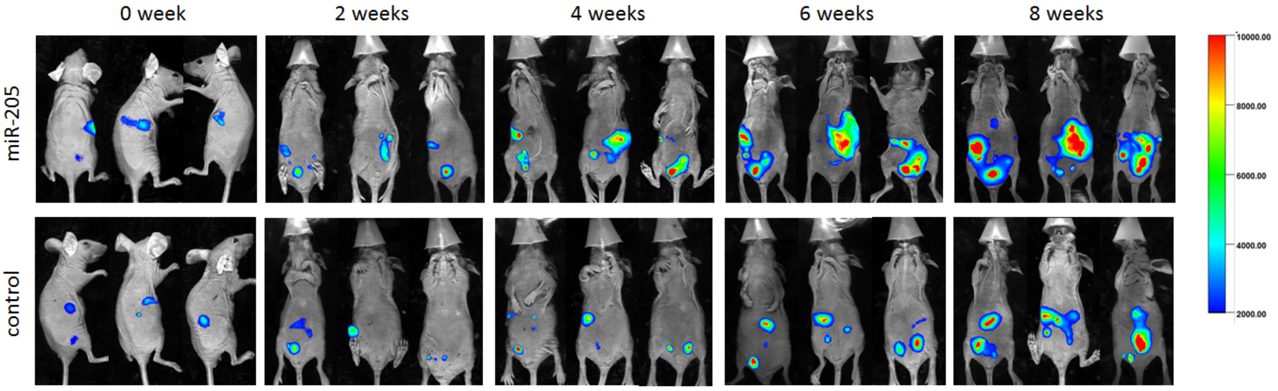

Supplementary Figure 2. miR-205 promotes OC tumorigenesis in animal model. Animal pictures illustrating the tumor growth in nude mice at different time points between 0 week and 8 weeks (from left to right) monitored by IVIS system in control (bottom) and tested (top) mice.

Supplementary Table 1 Primers and sequences used in this study

Gene	F/R	Primer sequences (5'— 3')
BMP6-F	F	ATCCCAGATTCCTGAGGGTGA
BMP6-R	R	CCCATACTACACGGGTGTCCA
SMAD4-F	F	CAGCTATGCCAGAAGCCAGA
SMAD4-R	R	GAACTCCTGGGACTTTCAACTGAC
PTPRJ-F	F	CCACCGTTTATTCCCAAGCA
PTPRJ-R	R	AGGCTTGTGGCACTGATGTTC
GCNT3-F	F	AAACAGAATCACGCCTTGTGAAGA
GCNT3-R	R	AGCCCAGAGCCCACAAGTAATG
QKI-F	F	GTAGGCACCAGCTGAAACTGAGTAA
QKI-R	R	CACAGATATCCACAGGAGCCAGA
PSD-F	F	CGCCACTTCACTCAGGCTTCTAC
PSD-R	R	GGACACCTGGATCCCTAAAGCA
DRAM1-F	F	AATCAAACCTGCTATTTCAGCACTC
DRAM1-R	R	CATGAACCACTGGCACAGCTA
ADAMTS9-F	F	CTCACGAGCACACCTGGAGA
ADAMTS9-R	R	TTAGGCTCATGGCCTGAAGA

ADAMTS18-F	F	ATAGGGCAACATGGACTGTTTAAGA
ADAMTS18-R	R	GGACTGATTCAGCAAGCACCAA
SMTM4-F	F	CCCAGATCAACTGGAATCTGAC
SMTM4-R	R	CCAGGAATGTGTTCACTGCATA
FAM32A-F	F	AAGGCATCCAAAACCCACAA
FAM32A-R	R	ATACCAAAGGAAACACACACAACC
SALL2-F	F	CACGAATCCGAGAGGAGCTCTC
SALL2-R	R	CACCATTACAGGAGGGTCAGTAG
SATB2-F	F	GGAGAACGACAGCGAGGAA
SATB2-R	R	CCGATGTATTGCTTTGCCTAGT
PTEN-F	F	ACGGGAAGACAAGTTCATGTAC
PTEN-R	R	TTTGACGGCTCCTCTACTGT
GAPDH-F	F	GCACCGTCAAGGCTGAGAAC
GAPDH-R	R	TGGTGAAGACGCCAGTGGA
